# Credit Default Swaps Drawup Networks: Too Interconnected to Be Stable?

**DOI:** 10.1371/journal.pone.0061815

**Published:** 2013-07-03

**Authors:** Rahul Kaushik, Stefano Battiston

**Affiliations:** Chair of Systems Design, ETH Zurich, Zurich, Switzerland; Universidad Veracruzana, Mexico

## Abstract

We analyse time series of CDS spreads for a set of major US and European institutions in a period overlapping the recent financial crisis. We extend the existing methodology of 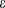-*drawdowns* to the one of *joint* 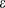-*drawups*, in order to estimate the conditional probabilities of spike-like co-movements among pairs of spreads. After correcting for randomness and finite size effects, we find that, depending on the period of time, 50% of the pairs or more exhibit high probabilities of joint drawups and the majority of spread series are trend-reinforced, i.e. drawups tend to be followed by drawups in the same series. We then carry out a network analysis by taking the probability of joint drawups as a proxy of financial dependencies among institutions. We introduce two novel centrality-like measures that offer insights on how both the systemic impact of each node as well as its vulnerability to other nodes' shocks evolve in time.

## Introduction

Within the field of complex networks [1], the investigation of financial networks is currently one of the emerging avenues [2], [3], also in view of the on-going global financial crisis.

A major issue concerns the assessment of the systemic importance of nodes, especially in the face of partial information on the network of dependencies. While financial contagion on networks [4]–[6] differs in some important respects from the epidemics spreading [7], [8], in both processes the topological structure of the network plays a crucial role in the collective dynamics and therefore in the emergence of systemic risk. A body of work focuses on networks reconstructed from correlations among equity prices or return time series [9]–12. For instance, the analysis of the minimum spanning tree provides insights into the classification of stocks and the level of correlation depending on the market phase. Correlation analysis suffers, however, from some important limitations, the main one being that zero correlation between two series does not imply that they are independent (only the inverse is true). To overcome these limitations, here we utilise a method based on the detection of *joint* 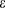-drawup, which allows us to estimate the probability that two series exhibit a co-movement. An 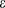-drawup is essentially a persistent upward movement in a time series until a peak has been reached, after which the time series experiences a decline (or, has a “correction”) that exceeds the amplitude 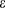 (see Methods). Moreover, in contrast to equities, CDS prices reflect the default probability of the reference entity and thus the network constructed from CDS prices are more relevant in studying the propagation of default risk.

**Figure 1:**
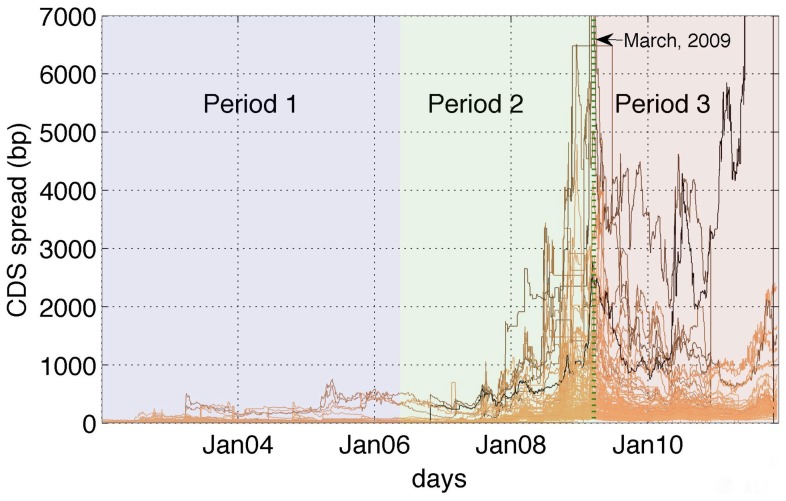
Time series of credit default swaps throughout the credit crisis. A plot of the CDS spread time series covering the financial crisis of 2008. The data ranges from January 2002 to December 2011. We can observe three market phases. Most CDS spreads peak around March 2009. The CDS prices are quoted in basis points (bp). The purpose of this plot is to highlight the market regimes, rather than the individual CDS spread evolution. Accordingly, the CDS spreads of all the financial entities are plotted here.

Our approach can be applied to construct networks of dependencies in other financial markets. In general, it applies to all domains of networks in which links are, for any reason, unobservable but the dynamics of the nodes reflect the dependency structure. To summarise, the contributions of the paper are the following. First, we build on the 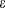-drawdown method [13] to estimate the probability of *joint* 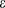-*drawups*, which are essentially a particular type of co-movements across time series. Based on this, we estimate the level of the so-called interdependence and trend reinforcement [14], [15], in the system across different phases of the market (see Figure 1). This finding is of interest in light of previous theoretical work on the emergence of systemic risk [16]. Second, we construct a network of interdependencies among institutions and we introduce two novel centrality measures that allow for the identification of systemically important nodes in the network. Our approach enables the disentanglement of a structure that is, only apparently, very homogenous. It also allows us to track how the role played by nodes in the network evolve in time.

## Results

### Interdependence and trend reinforcement

Whilst interdependence can be seen as a form of risk diversification which decreases individual risk, previous work [16] has demonstrated that high interdependence leads, instead, to higher systemic risk when coupled to a so-called trend reinforcement [14]. We thus proceed to investigating the presence of interdependence and trend-reinforcement in the CDS markets. In our context, trend reinforcement refers to the tendency of an 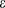-drawup to be followed by another 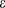-drawup in the same time series. Interdependence refers, in contrast, to the existence of co-movements between two different time series (i.e. an 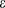-drawup followed by another one in a different time series). Here, we take the frequency of 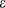-drawup's in 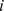 as an estimate of the probability 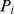 that security 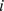 has an 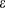-drawup. Similarly, for the frequency of *joint* 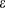-drawup's we estimate 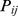, i.e. probability that 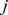 experiences an 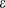-drawup given that 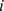 experiences an 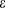-drawup. The expected probability of joint drawup's in the case of two statistically independent time series is 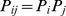. Therefore, we take as an estimate of interdependence between two financial institutions, the deviation from such a case, i.e. 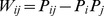. Finally, in order to account for finite size effects, we consider only those values of 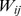 that cannot be rejected based on a permutation test and we reset to zero all the other values. In such test, each 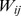 value is retained only if it is found to have less than 5% chance to come from a distribution obtained after permuting the position in time of all the 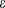-drawup's. For more details, see Correction for Randomness in Section Materials and Methods. In the following, we refer to this procedure when we say that values are statistically significant at a 95% confidence interval.

We also account for a time lag 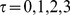 days between the drawup's and we take the average of 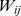 across 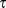 values. Analogously, we take as an estimate of the trend reinforcement for institution 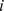 the deviation: 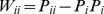 and we treat it as above. Notice that, because of the time lag 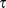, 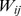 is not a symmetric matrix. Notice also that a positive value of 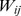 does not imply a causality relation between the movements of 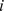 and 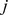, but measures the dependence of 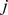 due from 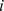 in terms of a conditional probability.

**Figure 2:**
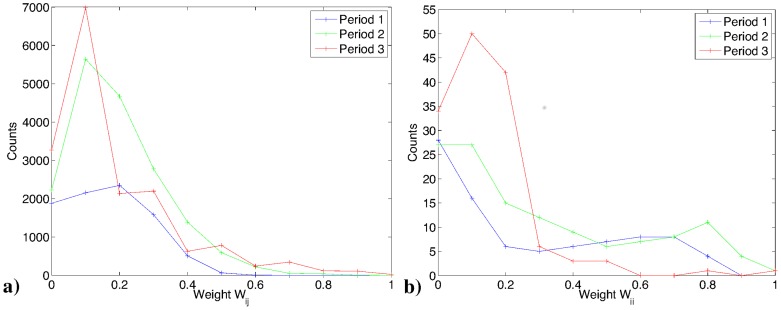
The distribution of non-zero values of interdependence 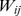 across the three periods. (a) The counts in periods 2 and 3 are higher than in period 1. In addition, during periods 2 and 3 the distributions of 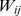 have longer tails compared to period 1. (b) The distribution of non-zero values of trend reinforcement 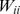 across the three periods.

The distribution of 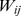
's and 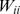
's are shown in Figure 2 a, b. The histograms count only the non-zero values of 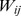
's and 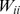
's (i.e., as explained above, only those that are found to be statistically significant at a 95% confidence interval, according to the permutation test. We find statistically significant levels of trend reinforcement, in about 50%, 72% and 80% of the nodes (respectively in period 1, 2 and 3). The range of values of 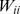 across periods 1, 2, and 3 are: 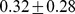, 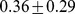, and 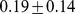 respectively. In Figure 2 b), the curve for period 2 and 3 is mostly above the one for period 1. This means that the number of nodes with a significant level of trend reinforcement increases when the market moves from the first phase to the following two, more volatile, phases. We also find statistically significant levels of interdependence in 54%, 78% and 77% of pairs of nodes in period 1, 2, 3, respectively. The range of values of 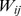 across periods 1, 2, and 3 are: 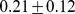, 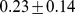, and 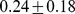, respectively. The histograms of 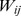 (see Figure 2 a) show that periods 2 and 3 are characterised by higher frequencies. In fact, 20% of pairs in period 2 and 19% pairs of nodes in period 3 exhibit values of 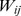 greater than the mean plus one standard deviation of period 1. Moreover, while in period 1, nearly all values of 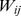 are smaller than 0.5, in period 2 and 3 there is a tail extending up to 1.

These findings show that interdependence and trend reinforcement are indeed present in an important market such as the one for CDS's. Moreover, trend reinforcement increases from period 1 to period 2, and even more so does interdependence.

### Network analysis and centrality

There is a growing body of works looking at CDS markets, and more in general at derivative markets, as networks in order to investigate the systemic importance of market players [17]. In light of the previous section, in our context The CDS market can be naturally mapped into a directed and weighted network in which nodes represent institutions and edges represent interdependencies among institutions. More precisely, whenever 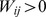 (recall that we have retained only the values that are statistically significant, see Materials and Methods), we assign a weighted edge with value 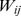 from institution 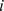 to 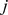. Since 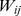 is the probability that conditional to 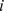 experiencing a draw-up, 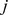 also experiences a draw-up with a time lag 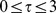, it follows that the stronger the edge, the stronger the impact that 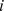 has on 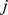. When looking at the properties of connectedness of the network, we find a significant number of disconnected nodes in all three periods (81, 39, 39, respectively). Remarkably, the rest of the nodes form only one strongly-connected component (LSCC, see Materials and Methods) encompassing, respectively, 95, 137, 137 nodes in period 1, 2 and 3. The density of links (i.e. the number of links over the number of possible links) in the LSCC is high in all the three periods: 0.98,0.97, 0.97. This is reflected also in the average out degree in the LSCC's across the three periods, which is 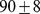, 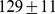, and 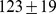. Finally, the average path length within the LSCC's is 1.04, 1.05 and 1.2, meaning that almost all the nodes in the LSCC are first neighbours to each other. In such a structure, each node has a direct impact on all the other nodes, and each of these has a further impact on all the others. Intuitively, this finding suggests that the financial distress at one node in the SCC can quickly propagate to all the other nodes in the LSCC and keeps reverberating through the many connections.

Centrality measures are used in order to understand the systemic impact of nodes in a financial network, e.g. DebtRank [6]. Here we want to focus on both the impact that a node makes on the others as well as on the impact that all the others make on it. The out- and in-degree of a node are the simplest measures of centrality that hold a valuable interpretation here: A high out-degree represents the ability of a node to affect many neighbours when it experiences a draw-up; a high in-degree corresponds to a node being affected by many nodes. Since the network is almost a complete graph, based on the out-degree, all nodes are equally systemically important and equally affected by the others. As an alternative approach, based on the notion of feedback centrality, for each node 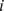 we introduce a novel measure, called *impact centrality* and denoted as 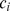, see Eqn. 1. The measure takes into account, in a recursive way, the fact that a node is more systemically important if it impacts many systemically important nodes (see Materials and Methods). Symmetrically, we also introduce the *vulnerability centrality* of a node 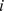, denoted as 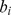, see Eqn. 2. This measure captures, instead, the idea that a node is more heavily vulnerable if it has strong dependencies from many nodes which are in turn heavily vulnerable. In both cases, the values are normalised between 0 and 1. In analogy to the random walker for PageRank [18], both measures hold a physical interpretation in terms of expected numbers of 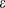-drawup's. The first is proportional to the expected number of 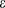-drawup's that occur in the network, conditional to a first 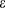-drawup at node 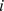. The second is proportional to the expected number of 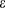-drawup's that occur in 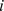, conditional to a first 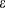-drawup occurring at all the other nodes.

**Figure 3:**
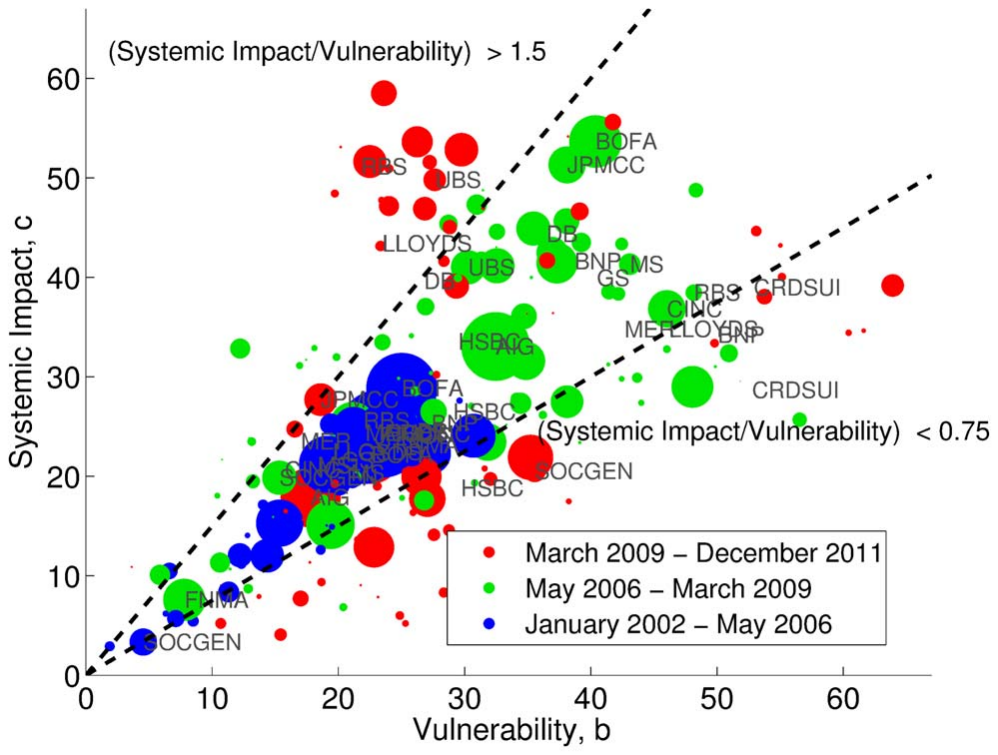
Scatter plot of impacting versus vulnerability centrality. Each institution in the CDS market is represented by three dots depending on the period (blue, green, red refers to period 1, 2, 3, respectively). The size of each node is determined by the average debt of a financial institution relative to the maximum average debt of a financial institution in a given period. Note that in this picture we present the non-normalised values of systemic impact and vulnerability, this exercise enables the comparison of node's centrality measures across periods. It can be seen that, while in period 1 most institutions are located between the two dotted lines, in period 2 and 3 many of them move to the top and bottom region. This means that ratio between the two centrality measures varies with the market phase. Few institutions of interest are labelled. For example, Bank of America (BOFA) remains in the same region across the three periods. With reference to the subsequent bow-tie construction used in Figure 4: The scatter plot is divided into 3 regions. Nodes in the region above the line 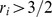 correspond to the IN. Nodes in the region 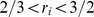 correspond to the SCC. Nodes in the region 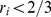 correspond to the OUT.

Figure 3 is a scatter plot of first-order impact centrality and first-order vulnerability centrality of each firm, i.e. taking into account only the immediate neighbours of each node (see Materials and Methods). Values of both centrality measures are not normalized here in order to compare them across different periods. The size of the circles reflects the average debt level of each firm. As we can see, some firms in period 1, eg. BOFA, exhibited a systemic impact of approximately 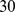. This can be interpreted as: an 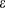-drawup in the CDS time series of a firm affects the CDS time series of an expected number of 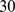 other market participants. In particular, there is a group of institutions with similar values of both impact and vulnerability between 20 and 30, which moreover also have the highest debt levels. This suggests that a perturbation in their debt levels would spread across a very large subset of the network. Since the size of the debt of such institutions is the largest, a small percentage change in their debt would cause a large change in the distress of the debt issued by others firms in the network. In period 2, both impact and vulnerability centrality tend to increase and almost double for many of the larger financial institutions, but this general increase is very heterogenous. In period 3, many firms decrease their debt, their impact and their vulnerability except a small group institutions for which impact remains high. Notice that the size, impact, and vulnerability centralities are not linearly correlated, e.g. UBS in period 3 exhibits high impact but low vulnerability, while at the same time, it experiences a drop in its total size.

The period under observation although covering 10 years represents a single observation of a crisis and cannot be used to draw definitive conclusions on the emergence of crises in general. However, recent works on systemic risk in financial networks have shown that the number of links play an ambiguous role. Few links are functional to diversify the individual risk. However, too many links generate systemic risk if they co-exist with mechanisms that either amplify the distress (such as in the case of contagion), or simply increase the persistency of the distress in time (such as for trend reinforcement) [16]. As we have seen, the CDS market exhibits a core of more than 100 nodes, that is almost a fully connected graph (i.e. with maximal degree) and where the weight of the links represent in many cases strong interdependencies. Moreover, the time series exhibit also a high level of trend reinforcement. Thus, according to the theoretical results mentioned earlier, in such a situation, even small levels of amplification can make the whole system very unstable. Notice that moving from period 1 to period 2, the values of most CDS's raised dramatically, in many cases by one order of magnitude (see Figure 1). This argument together with the finding described by Figure 3 suggests as a possible intuitive narrative that the CDS market was already potentially unstable in period 1, and that period 2 represents an unraveling that sooner or later would have happened anyway and that was mitigated by the intervention of the lender of last resort.

Notice that it is generally thought that without the massive intervention of the *Federal Reserve* (FED) through various emergency programs that lasted from the fall of 2008 until the summer of 2009, [19] there would have been a melt-down of the whole financial system. Indeed, previous findings based on different data [6] estimate that in that period the default of a few institutions would have triggered a systemic default.

### Link Pruning and Bow-tie Extraction

While in- and out-degree remain very homogenous over time and not very informative since the graph is very dense, the values of both centrality measures are more broadly spread across the range 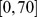 (see Figures S1, S2, S3 in File S1). If we focus on the ratio between impacting and vulnerability centrality, 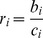, in the scatter plot of Figure 3, it is possible to identify three regions (above, between and below the dotted lines), corresponding to three different roles of the nodes.

Nodes are located in the top region if they have a value 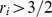, meaning that they impact the network 1.5 times more than they are vulnerable to it. Symmetrically, nodes are in the bottom region if 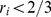. Finally, nodes that appear in the middle region are those that impact and are vulnerable to the network in a comparable manner. According to this classification, while in period 1 most institutions are located in the middle region, in period 2 and 3 they progressively move to the top and the bottom region. We observe that there are many nodes in the network that not only have a high impacting centrality, but also a high vulnerability centrality. From a systemic risk perspective it is essential to study nodes that are prone to distress; however, from a policymakers perspective it is also vital to monitor nodes that are not only prone to distress, but that also have a high impacting centrality as distress in such nodes could lead to a systemic collapse.

The values of *impact* and *vulnerability* centralities spread out from period 1 to period 2 and 3 (see Figures S1, S2, S3 in File S1). Thus, in order to visually enhance the role of nodes that are mostly one or the other we carry out the following link pruning procedure. In each period, for nodes located in the top region, we remove all their incoming links. Symmetrically, for those in the bottom region, we remove all the outgoing links. Since the initial network is strongly connected and dense, in this way, we obtain a bow-tie structure (see Materials and Methods). The position of a node in the bow-tie is related to its systemic importance. Indeed, the IN, SCC and OUT component of the bow-tie correspond to the top, middle and bottom regions of Figure 3, respectively (e.g. the nodes in the IN are those that impact the network more than they are vulnerable). Note that the *bow-tie* structure is constructed based on the choice of impacting-vulnerability centrality, i.e. nodes with 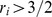 are in the IN, nodes with 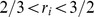 are in the SCC, and nodes with 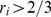 are in the OUT. In fact, for any 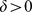, where 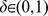. The lines 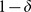 and 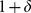 would separate the nodes into three regions. Thus, the choice of 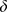 is based on the level of impacting-vulnerability centrality that is of interest. As an exercise to verify the effect of 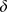 on emergence of a bow-tie structure in our network, we perform as robustness analysis on 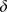 (see Figures S5, S6, S7, S8, S9, S10, S11 in File S1) for more visualisations. Notice that if a network is a directed SCC, and one truncates all incoming links of nodes with 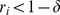, and all outgoing links for nodes with 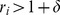. Then, it is not always the case that the filtered network has a non-trivial SCC (see Figure S4 in File S1).

**Figure 4:**
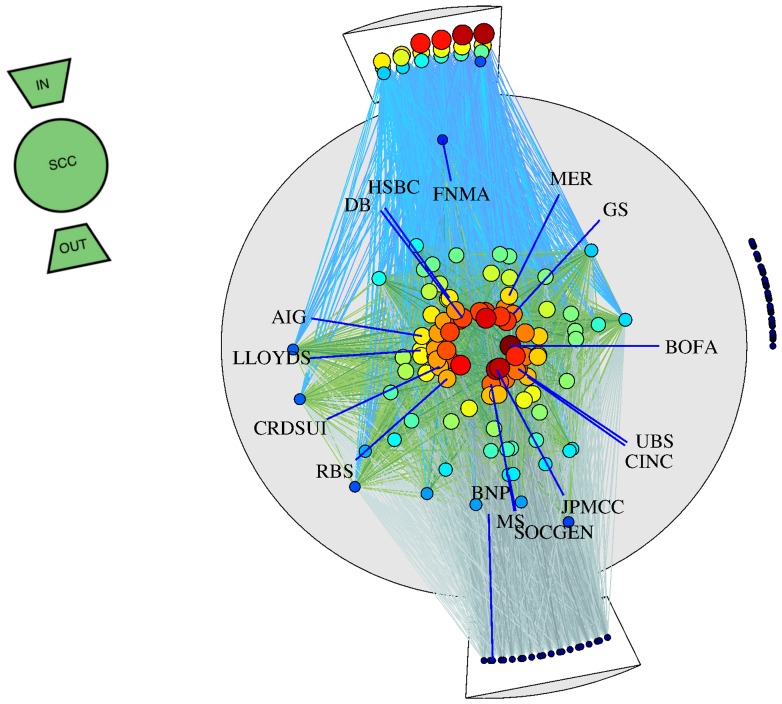
The network of the CDS reference entities from period 2. Each of the nodes represents a financial institution. Outgoing links from nodes that are in the top, or the IN of the bow-tie structure represent the estimated potential impact of a financial institution to its neighbours (see Materials and Methods). The nodes in the SCC are placed within a circle of radius one and centred at the origin. The distance of each node from the centre is 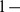
Impacting centrality. The angle increases linearly from 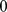 to 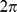. Thus, the closer a node is to the centre the higher is vulnerability-impacting centrality. Similarly, nodes in the OUT and IN are placed between angles 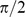
- 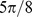 and 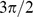 - 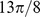 respectively. In addition, nodes in the OUT and IN are placed with an offset of 1.1 from the origin. With the bow-tie representation we are able to visually compare the centrality of a node 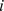 with node 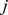. Also, with this visualisation we are able to extract a network of nodes that mostly impact the others, nodes that impact just as much as they get vulnerable, and nodes that are only vulnerable to the other nodes in the network. The size and the colour of the node reflects vulnerability-impacting centrality of a node (nodes with larger vulnerability-impacting centrality are in red). The colour assigned to links is based on where the links point to in the network. Links originating from IN to the SCC are in bright blue. Links originating in the SCC to nodes in the SCC are in green. Links that are originating in the SCC to the OUT are dull blue grey colour.

We then introduce a novel method for the visualisation of the bow-tie (Figure 4). This enables the representation, at the same time, of a network structure, the position of the nodes in the various component of the bow-tie, as well as their level of impacting centrality (see Table S2 in File S1). In Figure 4, the circle represents the SCC, the top (bottom) section correspond to the IN (OUT). E.g. within the SCC, more central nodes are located towards the centre of the circle. The colour code and the size of the dots also reflect their centrality, such that the red and large dots are the most central (see caption of Figure 4). This visualisation allows to track how individual institutions become more or less central, or if they changed role across periods (see File S1). In period 1, most of the nodes of the bow-tie are in the SCC (85), with 4 and 6 in the IN and OUT respectively. Moreover, most nodes in the centre of the SCC are banks and investment banks, while insurance and real estate companies tend to be in the periphery of the SCC (see File S1), This implies that in the normal phase most of the nodes impact the network, and are vulnerable to the network in a comparable manner. In period 2, the bow-tie grows overall, but the SCC (97 nodes) grows proportionally less than IN (19 nodes) and OUT (22 nodes). Of the 81 nodes that were disconnected in period 1, twenty seven migrated to the SCC (Figure 4). In period 3, the size of the bow-tie remains unchanged, but the SCC shrinks by about a 50% (from 97 to 47 nodes), as a result of a migration to the IN (37 nodes) and mostly to the OUT (53 nodes) (see Table S1 in File S1). In particular, the nodes with high impacting centrality are now all located in the IN and not, anymore, in the SCC.

One should not forget that the original network is a strongly connected graph and the bow-tie is obtained with a filtering. Therefore, it is not the case that the nodes in the IN are not connected among each other. This means that in case a few nodes would have defaulted, the others would still have been heavily affected. However, the observed migration of nodes implies that, compared to the normal period, there has been an increasing polarisation between nodes (IN) that predominantly impact the network, and nodes (OUT) that predominantly are vulnerable to the network.

The above analysis of impacting and vulnerability centralities, and the bow-tie extraction allows us to move from an initial picture in which all nodes seemed to be equally important from the point of view of systemic risk, to a much more refined picture. In terms of systemic impact, we can now focus on a small subset of the nodes, viz. nodes that have a high impacting centrality and are located in the centre of the SCC, or in the top part of the IN. This finding is corroborated by anecdotal evidence [20] about the role of important actors of the credit crisis of 2008 (see File S1). Conversely, the impacting centrality allows also to identify nodes that suffer the most from an impact originating from the others. Remarkably, there is no evidence of one or two nodes dominating the others in terms of systemic importance. In contrast, we see that in each period a set of about top 19 nodes have similar values of centrality.

## Materials and Methods

### Data

**Figure 5:**
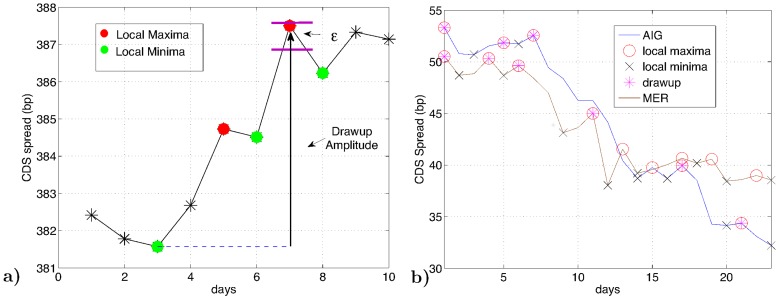
Illustration of the 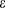-drawup methodology. (a) The * represents local extrema that were not detected as drawups. The red-dots represent local maxima that were picked up as candidates for a 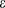-drawup. The green-dots represent the local minima that were picked up for a 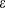-drawup. Compare the difference between the maxima and minima on days 5 and 6 respectively with 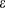. Since, 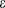 greater than the difference, we iterate to the next set of local maxima and minima on days 7 and 8, keeping day 3 as the day from when we count a 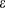-drawup (b) The plot highlights the 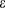-drawup methodology applied to the time series of American International Group (AIG) and Merrill Lynch (MER).

*Credit Default Swaps* (CDS's) are financial derivatives instruments in which the seller provides the buyer protection against a credit event of a reference entity (see File S1). Our aim is to analyse the time series data of CDS prices, or spreads, of top US and European financial institutions in the last years. The data, acquired via a subscription to Bloomberg, consists of CDS spreads of single name entities denominated in US dollars and in the Euro, encompassing a total of 176 top firms in the financial sector, in the period from 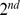 January 2002 until 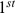 December 2011. As shown in Figure 1, the time series display three distinct phases. Accordingly, we divide the data into three parts: (1) January 2002–May 2006 (representative of a normal phase); (2) May 2006–March 2009 (volatile with an upwards trend); (3) March 2009–December 2011 (volatile with a downwards trend market scenario). The motivation to do a period-wise analysis is to extract the network structure before, during and after the crisis of 2008. This data window covers a 2560 weekdays. 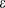-**drawups**. For each institution's time series we detect what we call 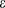-drawup's. An 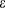-drawup is an extension of the notion of an 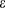-drawdown [21]. It refers to a persistent upward movement in a time series until a peak has been reached, after which the time series declines (or, has a “correction”) by more than an amplitude 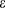 (see Figure 5a). Since the CDS spread represents the cost of insurance, an 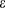-drawup signifies an increase in the default probability of that institution, as perceived by the market.

We compute the 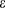-drawup's in each of the time series using the following algorithm, which we describe using the example illustrated in Figure 5a. Suppose, we start our analysis of an 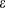-drawup from the first green point from the left in Figure 5a. The step are as follows: (1) We compute the local variation in the time series for the last ten days, call it 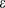. (2) compute local extrema. (3) Goto first local minima, call it 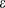-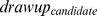 (on day three, Figure 5a). (4) Iterate to the set of local maxima and minima (occurring on days 5 and 6 respectively, see Figure 5a). Compute the difference between the maxima and the minima, call it *correction amplitude* (*correction amplitude* refers to the decline in price followed after an increase in price). (5) Update 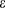 by computing the local variation of the last ten days. (6) If *correction amplitude* 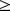 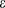. Then, we record the 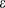-drawup on the day it occurs (day 7 in Figure 5a). And, we update 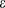-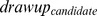. Otherwise, we iterate to the next minima and goto the succeeding maxima and repeat steps above.

The choice of using 10 days to compute local variations was the result of following preliminary analysis. We have computed drawups for 50 time series using various numbers of days ranging from 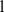 until 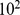. At one extreme we take all the local maxima in the time series as drawups, which is not desirable (see Figure 5b). At the other extreme the algorithm ignores too many drawups. 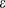-drawups in general can be validated by eye only and thus we could not run an optimisation function that would maximise the number of “true” drawups, as a function of the number of days chosen to compute local variations. After a thorough visual inspection of 50 time series at various scales, we picked 10 days as the best choice of the time window. Note that on weekends and holidays, the last traded price is carried forward; however, we have verified that this does not affect the 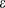 - drawup algorithm.

With the above procedure, we are able to detect the 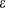-drawup's in the the time series data. Once we have detected the 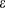-drawup's in 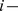
th time series, we construct the vector 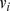 for node 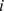, whose length is the same as the length of the time series, 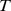. Also, 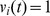 if there was a drawup on day 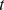 in node 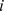, and zero otherwise.

#### Co-movements

When market participants buy and sell insurance on each other, their financial performances can become interdependent (see File S1). Therefore, we are interested in detecting joint upward movements in pairs of time series. In order to detect co-movements we implement the following algorithm (notice, that the resulting matrices of co-movements 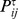 are square but not symmetric):

(1) Select a given node 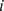. (2) Loop from day 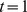 till 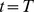 and compare each 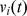 with all 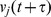 where 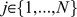 and 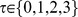. (3) If 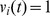 and 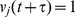, then 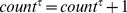. (4) Update a matrix of counts of joint drawup's, i.e. 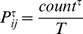. (5) Repeat the steps (1)–(4) for all node 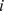.

#### Correction for Randomness and Finite Size: Statistical Significance

In order to account for the co-movements that could arise by chance, for each pair 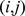 we subtract the expected number of co-movements in the case of independent events and we obtain 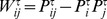. In order to correct for finite size effects, we carry out a permutation test. For each pair 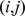 we generate 100 permutations of the respective time series of 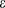-drawups. We compute the corresponding 100 values of 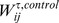 and the value 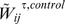 that corresponds to a 95% confidence level. This means that if the empirical value 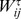 exceeds 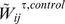, it has less than 5% chance to come from the same distribution. Accordingly, we keep the empirical value of 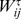 only if it passes this test and otherwise we set it to 0. In the following, when we say that only *statistically significant* links are retained, we refer to the above filtering procedure.

#### Interpreting the Conditional Probability Matrix W

We now average across values of 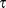 the filtered matrices, i.e. 
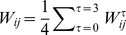
. Notice that the quantity represented by 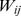 is not a measure of causality. However, under the assumption that the observed joint 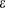-drawup frequencies are an approximation of probabilities, each entry 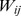 of the matrix 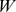 has a precise meaning: It is an estimate of the probability of an 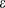-drawup in the time series of 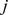 at a given day, conditional to an 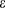-drawup in time series of node 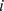 in the preceding 3 days and in the same day, averaged over the days of the time delay.

#### Impacting and Vulnerability Centrality

In line with the notion of feedback centrality (e.g., PageRank [18], see File S1), we introduce the Impacting Centrality 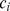,
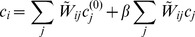
(1)and the Vulnerability Centrality 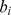,
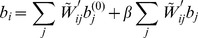
(2)In the definition above: 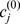 and 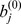 are the intrinsic centrality, which for the sake of simplicity are set to 1; the matrices are normalized so to be row-stochastic, 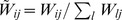, 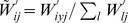, with 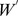 denoting the transpose of 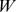; the parameter 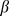 is a dampening factor which we set to 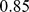 in line with the PageRank [18] heuristic [18]. Eqn. 1 and 2 can be analytically solved to yield the solution, 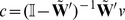 and 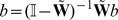. We can interpret the Impacting Centrality 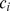 as the extent to which a firm 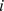 impacts the network via direct connections and, recursively, via indirect connections. Analogously, we can interpret the Vulnerability Centrality 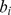 as the extent to which a firm 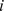 gets impacted by the network via direct connections and, recursively, via indirect connections.

In terms of physical analogy, in the case of PageRank [18], it is known that the score of a node is proportional to the expected number of visits of a random walker that is let free to navigate in the network hopping randomly from a node to the successor nodes. Notice that because of possible cycles in the network a walker can visit a node many times and thus the expected number of visits by random walkers can in general exceed the number of walkers. We can map the visit of the random walker into the occurrence of an 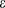-drawup. The Impacting Centrality of a node 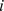 is then proportional to the expected number of 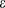-drawup's occurring across all nodes in the network, conditional to an initial 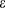-drawup at node 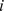. Conversely, the Vulnerability Centrality of a node 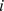 is proportional to the expected number of 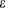-drawup's occurring at node 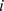, conditional to an initial 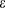-drawup at some node 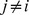 in the network.

#### Bow-tie structure

A bow-tie network is a directed network consisting of four main parts, as follows. The Strongly Connected Component (SCC): set of nodes such that each can reach any other via a directed path; OUT: set of all nodes that can be reached, directly or indirectly, from the SCC; IN: the set of all nodes that reach the SCC directly or indirectly. The fourth and last component of the bow-tie structure, Tubes and Tendrils (TT) represent the set of all nodes that are not a part of the SCC; however, a node in the TT can either be reached from the IN and/or OUT.

#### Link Pruning and Bow-tie Extraction

If a network is dense and strongly connected it is difficult to understand who impacts whom. We then proceed to the following link pruning. We compute the ratio between the Impacting and the Vulnerability Centrality, 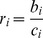, see Eqn. 1 & 2. If 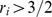 then we remove all the incoming links of the node 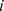. This means that all nodes that exhibit 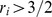 will only have outgoing links after the pruning. Similarly, nodes that exhibit 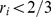 get all their outgoing links removed. The remaining nodes, i.e. such that 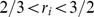 retain both the incoming and outgoing links. With few exceptions, this link pruning procedure extracts out of a dense strongly connected network a subnetwork with a bow-tie structure. This is useful to highlight the role of a node. Those nodes mainly impacting the others end up in the IN component, after the pruning. Those nodes mainly vulnerable to the others end up in the OUT. Those nodes being equivalently impacting and vulnerable end up in the SCC.

## Conclusion

We have analysed the 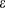-drawup's in the CDS's time series for the top US and EU institutions throughout the last 10 years. By measuring the frequency of joint drawup's in pairs of CDS time series we have estimated the level of interdependence and trend reinforcement in the market. According to previous theoretical works on financial networks, the interplay of these two mechanisms is deeply linked to the emergence of systemic risk. We have found statistically significant levels of both interdependence and trend reinforcement. Moreover, we see an increase of both in acute phases of the crisis. The result suggests that high interdependence and trend reinforcement together with high level of individual riskiness are possible indicator of the level of systemic risk. Indeed, when CDS spreads were at their peak in 2008, implying high risk of individual default, movements in the spread of a few institutions were very likely to be followed by movements in another and *also in the same* institutions. This means distress in a few key players would have likely propagated to many other players in the market.

Furthermore, we have carried out what to our knowledge is the first study of the complex network of CDS interdependencies. In order to investigate the systemic importance of individual nodes, we have introduced two novel measures. The impacting centrality captures, in a recursive way, how much a node impacts the network. Symmetrically, the Vulnerability centrality captures how much a node is vulnerable to the network. These two measures enable the extraction of a bow-tie structure from the initial network and to clarify the role of the nodes. In Basel III [22] interconnectedness has been identified as one of the pillars to identify Systemically Important Financial Institutions (SIFI). The interconnectedness of an institution can be assessed by its ability to impact other institutions and its vulnerability to the others in the financial network. We show that in the CDS markets size, impact, and vulnerability are not trivially correlated, at least not in the volatile phase of the market, i.e. period 2. In the initial phase the system is homogeneous with similar impact and vulnerability centralities across players, while in the following periods there is an increase in both impact and vulnerability centralities, but they do increase in a heterogenous manner.

The specific findings of this analysis are relevant to the broad audience interested in the issue of systemic risk and systemically important financial institutions, including policy makers. Moreover, our approach is very general and applies to any set of time series associated to units that operate in interaction. In particular, it is of interest for those cases where the direct interaction between units is not observable and the dependence has to be inferred from the dynamics. In this respect, our paper contributes to a stream of work on the observability and the reconstruction of complex networks [23] .

## Supporting Information

File S1 **Supporting information including Table S1, Table S2, and Figures S1, S2, S3, S4, S5, S6, S7, S8, S9, S10, S11, S12.**
(PDF)
