# Supplementary material for: Credit Default Swaps Drawup Networks: Too Interconnected to Be Stable?
Source: PLoS One. 2013 Jul 3;8(7):e61815. doi: 10.1371/journal.pone.0061815 (PMC3700991; doi:10.1371/journal.pone.0061815)
Supplement: File S1 — Supporting information including Table S1, Table S2, and Figures S1, S2, S3, S4, S5, S6, S7, S8, S9, S10, S11, S12. (PDF) [file pone.0061815.s001.pdf]

Supplementary Information: Credit Default Swaps Drawup Networks: Too Interconnected To Be Stable?

Rahul Kaushik[1], Stefano Battiston[2],
1 Rahul Kaushik Chair of Systems Design, ETH Zurich, Switzerland
2 Stefano Battiston Chair of Systems Design, ETH Zurich, Switzerland
∗ E-mail: Corresponding rkaushik@alumni.ethz.ch

Background Information on CDS

A CDS is a bilateral Over the Counter (OTC) derivative. It is analogous to an insurance instrument. A CDS contract has three legs, i.e., there are three entities that formulate a CDS contract. The three parties involved in a typical CDS contract are the buyer, seller and reference entity. The buyer of a CDS contract purchases protection on the reference entity from the seller for fixed periodic payments, also called premiums. It is not required that a buyer of a CDS contract have a credit exposure to a reference on which it is buying a CDS contract. Analogously, it is equivalent to saying that one can buy an insurance on one's neighbours house.

In the event that the reference entity defaults on its debt to its investors, the buyer receives a one time payment from the seller, hence the name *Credit Default Swap*. CDS's can be one of the following types: single name, Index and Basket CDS. As the names might suggest, single name CDS are on a single entity, sovereign or otherwise. Index CDS's are issued on constituents of an index with equal weights being assigned to each of the constituents. Basket CDS's can have more than one reference entity, in fact a basket of entities constitute a set of reference entities.

In the case of basket CDS's there are three further classifications, namely first-to-default CDS, full-basket CDS, untranched basket and a tranched basket, or Collateralised Debt Obligations (CDO). In addition, CDS contracts are negotiated privately and are bilateral in nature.

Like a swap agreement there is no initial payment that is needed to enter into a CDS contract. Unlike a corporate bond, a CDS contract enables a participant to go short in the credit of a reference entity. Also, one can enter into a CDS contract even if a corporate bond of some pre-specified maturity is not available.

The popularity of the CDS market can be seem from the growth of such products. International Swap and Derivatives Association (ISDA) statistics show that by the end of 2003, the notional amounts outstanding in the CDS markets stood at \$3.58 trillion dollars. Compared to the year 2000, in the year 2003 the CDS markets gained 1.4% percent of the entire swap market. The popularity of CDS contracts declined in the years 2009 and 2010. This was a fallout for the financial crisis of 2008. From Fig. S12, we can see that the CDS spreads were reflecting such movements in the market.

The embedded relationships that lie within a CDS contract remain opaque to the general public due to the fact that they are not only privately negotiated; but, also because information on such contracts exposes the financial institutions to corporate attacks. In addition, even if a comprehensive dataset were available, the lack of transparency with regards to ownerships leads to different level of complexity, see [1].

Data set

Bloomberg has negotiated contracts with various quote providers to display data on the Bloomberg terminal; however, not all quote providers relax the constraint of Bloomberg being allowed to distribute this data to the end user. Thus, we see certain breaks in our time series, as on those days either the CDS was not traded or is quoted by a quote provider that does not allow its data to be downloaded via Bloomberg. To circumvent this issue, we carry forward the last traded price till a time that a new price has been reported. Thus, CDS time series data were polished beforehand to make the analysis in the ε-drawups framework possible. Not all time series exist in the same time window, thus we take the CDS contract with the largest number of observations and use it as a reference of our time window. Thus, all time series are put in one matrix, where if a CDS time series did not exist whence the reference existed, then we assign a value of zero to it. This modification does not affect the dynamics of the CDS's per

se, as a spread of zero implies an absence of a swap contract anyways. Once, we have the data in a single matrix, we then iterate through all the individual time series and compute all the local extrema (see Methods). The date of each extrema is also recorded. We then proceed to computing ε-drawups. The ε parameter is a local and dynamic parameter. It is essentially the local variation of a time series in the last ten days. We compute the ε-drawups for each security and record the date at which the ε-drawup occurred. We then proceed to computing co-drawups pairwise. To compute common drawups we divide the dataset into three periods (see Methods in the main paper) and compute common drawups and the drawups experienced by each of the time series in that period. We also compute co-drawups for all the pairs with a time delay factor τ, i.e., when one security is translated by τ days w.r.t. another. We do this exercise for all pairs in our dataset. Finally, we compute w_{ij}'s (as before) using our count matrices.

Control Set

After having computed a matrix of ε-drawup's, for each security, we permute the matrix indices of where the ε-drawup's occur. This way we are re-arranging all the occurrences of ε-drawup's in a random manner. The reason to pursue this methodology and not generating random (or even , trend reinforced random walks) is that the authors don't wish to define the the price process as a priori, assuming random walks (or, trend reinforced random walks) are a good proxy for a CDS price process. In addition, we do not resample the original time series, as such a procedure introduces price movements that sometimes amplify ε-drawup's when there in fact are none. This key point becomes even more important when we want to develop a control set for all three periods. We perform a permutation test to filter the empirical $W'_{ij}s$. We compute W'_{ij} for each pairs of securities. To do this, we proceed with permuting the ε-drawups in each of the securities and compute W'_{ij}. We repeat this procedure a hundred times. With the hundred realisations of W'_{ij} for each pair of securities i, j, we then further filter W'_{ij} at the 95% confidence interval to derive a single number W^*_{ij} for each pair of securities. We then utilise W^*_{ij} as the control number to filter empirical W_{ij}, i.e., each empirical pair is filtered with a unique number that corresponds to the control number generated from our permutation test.

PageRank and Impacting Centrality

To compute the centrality of the nodes in the network, we take inspiration from the concept of PageRank that was introduced in the context of the World Wide Web (WWW) to enhance users' search experience [2]. The main theme of the idea revolved around determining the rank of a webpage based upon how many sites (other than itself) point towards it. Such a rank could be used as a good proxy for determining a webpages' relevance to user searches. Suppose, that the PageRank of each website, i, be denoted as C_i. Then, C_i for websites can be defined in a network framework consisting of N vertices. Consider,

$$C_i = \underbrace{\alpha \sum_{j \to i} \frac{C_j}{k_j^o}}_{\text{term 1}} + \underbrace{(1-\alpha)\frac{1}{N}}_{\text{term 2}} . \tag{1}$$

The α in term 1 on the r.h.s. of equation 1 represents the probability of C_j inherited by webpages j that are pointing to webpages i. Each webpage j contributes, proportionally $\frac{C_j}{k_j^o}$, to the webpage it points points to. Term 2 in 1 uniformly assigns the contribution of each of the webpages j to i times the complimentary probability from term 1. Unlike our measure of centrality, i.e., vulnerability-impacting centrality, PageRank centrality [2] measure is row stochastic. vulnerability-impacting centrality is analogous to the cumulative distress in the network on account of distress in node i.

The Bow-tie Structure & FCIC Report

A bowtie structure refers to a directed graph here, where the connected nodes are in one of the three parts of the network: IN (nodes with outgoing links only), SCC (nodes with both incoming and outgoing links) and OUT (nodes with incoming links). The resulting structure resembles that of a bow-tie where

the SCC occupies the position of a knot and the IN and OUT represent the respective wings of the tie. It is important to remember that the bow-tie structure is a construction that largely depends on the thresholds that are imposed upon the impacting-vulnerability centrality. We present the bow-tie structures from periods 1 until 3. The nodes are in the IN, if $r_i > 3/2$, see Fig. S1b, S2b, & S3b. The nodes in the SCC have $2/3 < r_i < 3/2$, and nodes are in the OUT, if $r_i < 2/3$. We also present the distributions of in-degree, out-degree, impacting, and vulnerability centralities for all three periods. We find that even though in-degree and out-degree have mass of their distributions in a narrow range; the impacting and vulnerability centralities across the three periods are distributed across a wider spectrum. Additionally, we present bow-tie structures from all three periods with varying degrees of thresholding, see Fig. S6, S7, S8, S9, S10, S11, and S5. The existence of the bow-tie structure is not guaranteed in all graphs. Consider, Fig. S4a & b. With these counterexamples we highlight that the existence of a bow-tie structure is not assured after the separation of nodes based on their level of impacting-vulnerability centrality. We find that in our network there is an SCC in all three periods after filtering the impacting-vulnerability centrality.

The Financial Crisis Inquiry Commission (FCIC) was established under the Fraud Enforcement and Recovery Act (Public Law 111-21) and was later passed by the Congress and officially signed and implemented by the President of the US in the month of May, 2009.

The goals of the FCIC was to examine the causes that led to the financial and economic crisis of 2008. During their investigation the FCIC conducted more that 700 witnesses and reviewed millions of documents. In addition, it also held public hearings in New York, Washington D.C. among other regions in the US. FCIC conducted extensive case studies on firms that it deemed as pivotal in bringing about the crisis. These firms include: American International Group (AIG), Bear Stearns, Citigroup, Countrywide Financial, Fannie Mae, Goldman Sachs, Lehman Brothers, Merrill Lynch, Moody's and Wachovia.

We present brief snapshots from the FCIC report [3] of some of the firms that the FCIC indicated were pivotal in financial crisis of 2008 along with the bow-tie visualisations from each of the three periods, see Fig. S1b, S2b, & S3b. In addition we also present the degree distributions for all three periods, Fig. S1a, S2a, & S3a. We also present some statistics on the distributions of nodes in the various regions of the *bow-tie* structure, see table S1, and the movement of some pivotal firms across the *bow-tie* structure in the three periods, see table S2.

	Impacting	Vulnerability	Both
Period 1	6	4	85
Period 2	22	19	97
Period 3	53	37	47

Table S1. Breakdown of nodes by region

	Period 1	Period 2	Period 3
AIG	SCC	SCC	OUT
GS	SCC	SCC	SCC (periphery)
BOFA	SCC	SCC	SCC (periphery)
CINC	SCC	SCC	SCC (periphery)
JPMCC	SCC	SCC	IN

Table S2. Location of AIG, GS, BOFA, CINC & JPMCC across the three periods in the *bow-tie* structure

AIG:

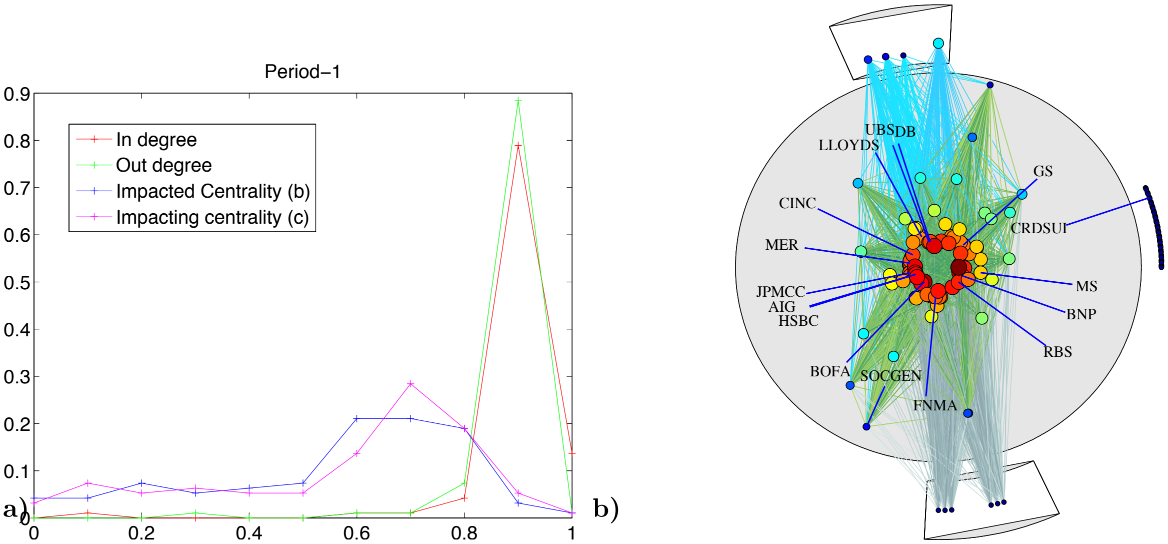

Figure S1. a) The normalised distribution of in-degree, out-degree, impacting and vulnerability centralities in period 1 . The bulk of the in-degree and out-degree distributions are concentrated in a narrow range. Impacting and vulnerability centrality distributions are distributed across the x-axis. **b) The network of the CDS reference entities from period 1**. Each of the nodes represents a financial institution. Outgoing links from nodes that are in the top, or the IN of the bow-tie structure represent the estimated potential impact of a financial institution to its neighbours (see Methods). The nodes in the SCC are placed within a circle of radius one and centred at the origin. The distance of each node from the centre is 1−Impacting centrality. The angle increases linearly from 0 to 2π. Thus, the closer a node is to the centre the higher is vulnerability-impacting centrality. Similarly, nodes in the OUT and IN are placed between angles $\pi/2$- $5\pi/8$ and $3\pi/2$ - $13\pi/8$ respectively. In addition, nodes in the OUT and IN are placed with an offset of 1.1 from the origin. With the bow-tie representation we are able to visually compare the centrality of a node i with node$j$. Also, with this visualisation we are able to extract a network of nodes that mostly impact the others, nodes that impact just as much as they are vulnerable, and nodes that only are vulnerable to other nodes in the network. The size and the colour of the node reflects vulnerability-impacting centrality of a node (nodes with larger vulnerability-impacting centrality are in red). The colour assigned to links is based on where the links point to in the network. Links originating from IN to the SCC are in bright blue. Links originating in the SCC to nodes in the SCC are in green. Links that are originating in the SCC to the OUT are dull blue grey colour.

- By 2005, AIG had written \$107 billion in CDS for such regulatory capital benefits; most were with European banks for a variety of asset types. That total would rise to \$379 billion by 2007. The same advantages could be enjoyed by banks in the United States, where regulators had introduced similar capital standards for banks' holdings of mortgage-backed securities and other investments under the Recourse Rule in 2001. So a credit default swap with AIG could also lower American banks' capital requirements. In 2004 and 2005, AIG sold protection on super-senior CDO tranches valued at \$54 billion, up from just \$2 billion in 2003. *FCIC Report*, page 140.
- AIG's business of offering credit protection on assets of many sorts, including mortgage-backed securities and CDOs, grew from \$20 billion in 2002 to \$211 billion in 2005 and \$533 billion in 2007, *FCIC Report*, page 141.

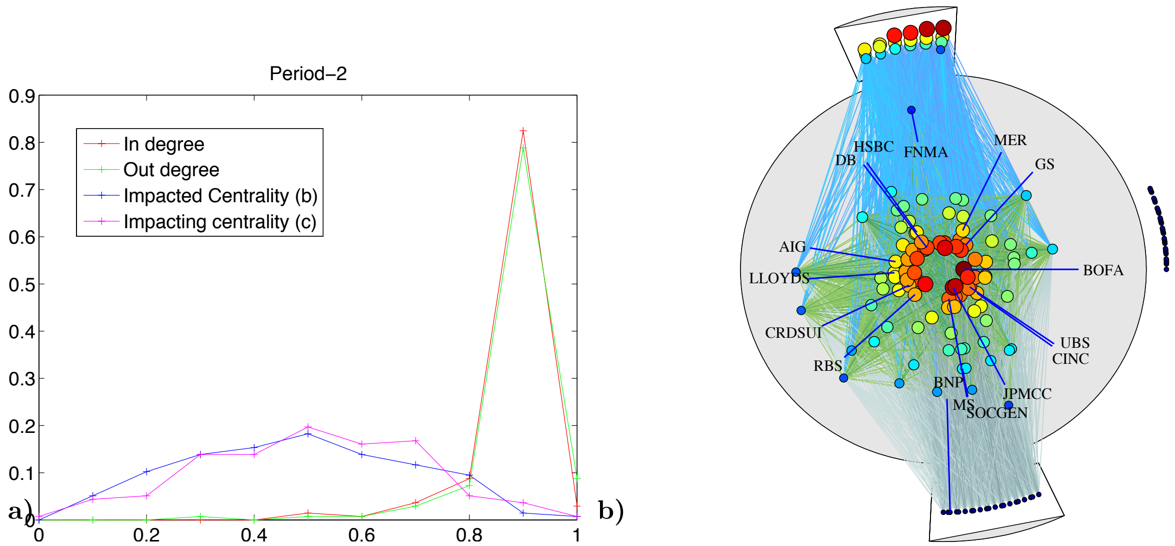

Figure S2. a) The normalised distribution of in-degree, out-degree, impacting and vulnerability centralities in period 2 . The bulk of the in-degree and out-degree distributions are concentrated in a narrow range. Impacting and vulnerability centrality distributions are distributed across the x-axis. **b)The network of the CDS reference entities from period 2**. The bow-tie is constructed as described in fig. S1.

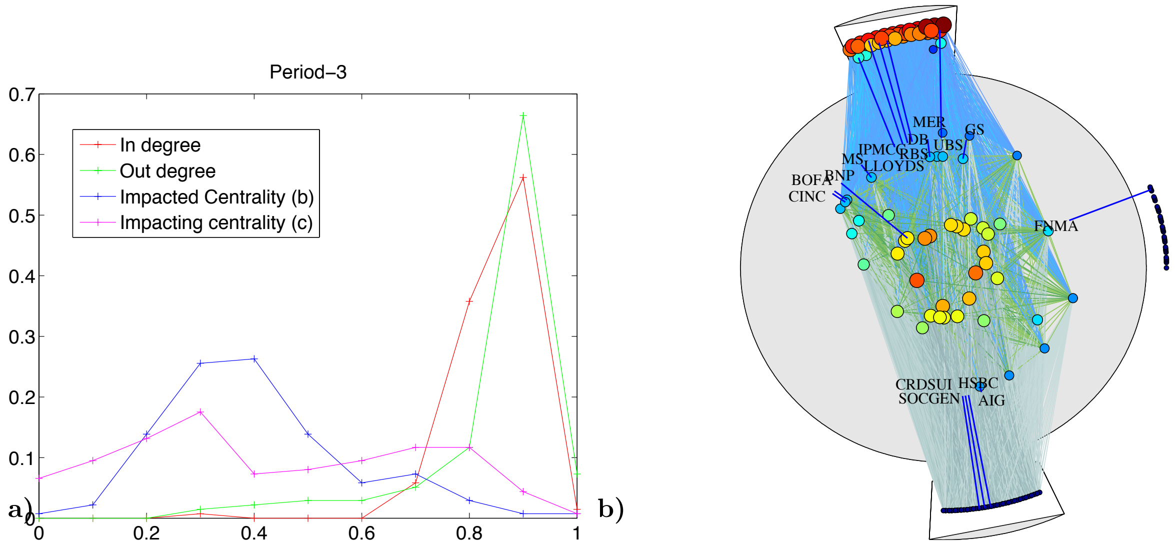

Figure S3. a) The normalised distribution of in-degree, out-degree, impacting and vulnerability centralities in period 3 . The bulk of the in-degree and out-degree distributions are concentrated in a narrow range. Impacting and vulnerability centrality distributions are distributed across the x-axis. Unlike periods 1 & 2, the in-degree and out-degree distributions are less peaked. **b)The network of the CDS reference entities from period 3**. The bow-tie is constructed as described in fig. S1.

GS

- Second, CDS were essential to the creation of synthetic CDOs. These synthetic CDOs were merely bets on the performance of real mortgage-related securities. They amplified the losses from the collapse of the housing bubble by allowing multiple bets on the same securities and helped spread them throughout the financial system. Goldman Sachs alone packaged and sold $73 billion in synthetic CDOs from July 1, 2004, to May 31, 2007. Synthetic CDOs created by Goldman referenced more than 3,400 mortgage securities, and 610 of them were referenced at least twice. This is apart from how many times these securities may have been referenced in synthetic CDOs created by other firms. *FCIC Report*, Conclusions.

- Goldman Sachs estimated that between 25% and 35% of its revenues from 2006 through 2009 were generated by derivatives, including 70% to 75% of the firm's commodities business, and half or more of its interest rate and currencies business. From May 2007 through November 2008, $133 billion, or 86%, of the $155 billion of trades made by Goldman's mortgage department were derivative transactions. *FCIC Report*, pages 51-52.

- Goldman's assets grew from $250 billion in 1999 to $1.1 trillion by 2007, an annual growth rate of 21%, *FCIC Report*, page 65.

BOFA, CINC, JPMCC

- From 1998 to 2007, the combined assets of the five largest U.S. banks: Bank of America, Citigroup, JP Morgan, Wachovia, and Wells Fargo more than tripled, from $2.2 trillion to $6.8 trillion.

- Leverage: Bank of America's leverage rose from 18:1 in 2000 to 27:1 in 2007. Citigroup's leverage increased from 18:1 to 22:1, then shot up to 32:1 by the end of 2007. *FCIC Report*, page 65.

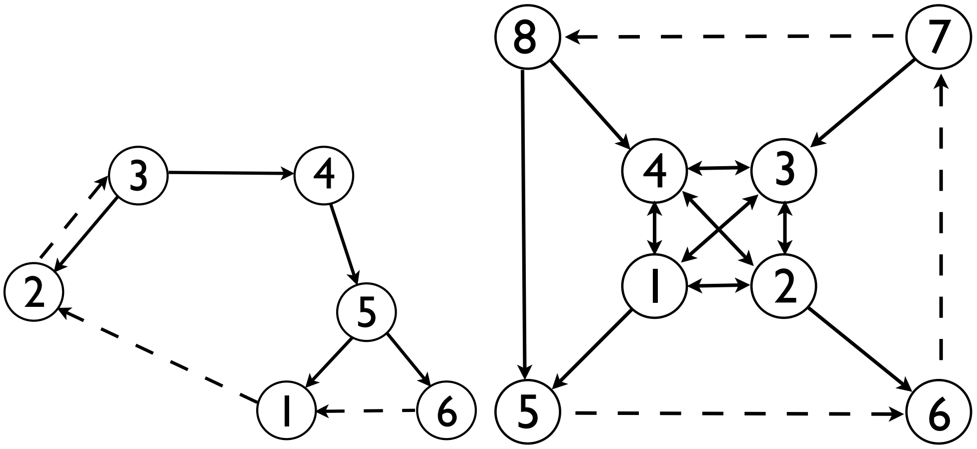

a) b)

Figure S4. a) Network W with an SCC: Dashed links are links that have been removed on the condition that $r_i < \theta$, where θ is some threshold. Then, we see that W no longer consists an SCC. **b) Network W with an SCC:** Dashed lines are as in **a)**. Then we see that, W still has an SCC. In fact nodes 1,2,3, and 4 remain in the SCC (as before) even after filtering links.

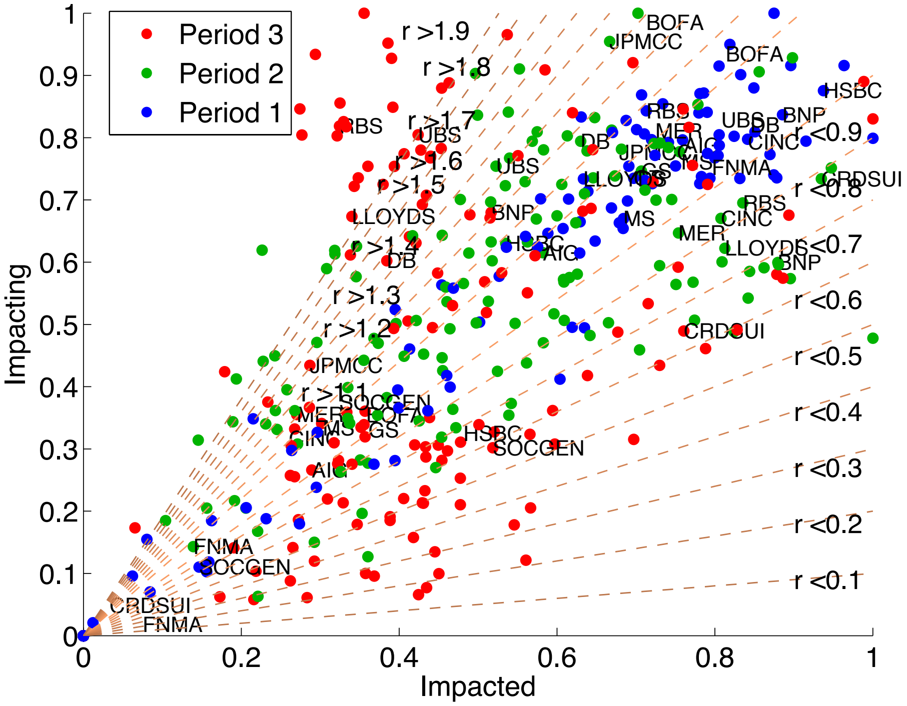

Figure S5. Scatter plot of impacting versus vulnerability centrality. Each institution in the CDS market is represented by three dots depending on the period (blue, green, red refers to period 1, 2, 3, respectively). It can be seen that, while in period 1 most institutions are located between the two dotted lines, in period 2 and 3 many of them move to the top and bottom region. This means that ratio between the two centrality measures varies with the market phase. Few institutions of interest are labelled. For example, Bank of America (BOFA) remains in the same region across the three periods. With reference to the subsequent bow-tie construction used in Fig. S6, S7, S8, S9, S10, S11, and S5: The scatter plot is divided into 3 regions for each choice of $\delta \in \{0.1, 0.2, 0.3, 0.4, 0.5, 0.6, 0.7, 0.8, 0.9\}$ where the upper and lower regions are given by $1+\delta$, and $1-\delta$ respectively. Nodes in the region above the line $r_i > 1+\delta$ correspond to the IN. Nodes in the region $1-\delta < r_i < 1+\delta$ correspond to the SCC. Nodes in the region $r_i < 1-\delta$ correspond to the OUT.

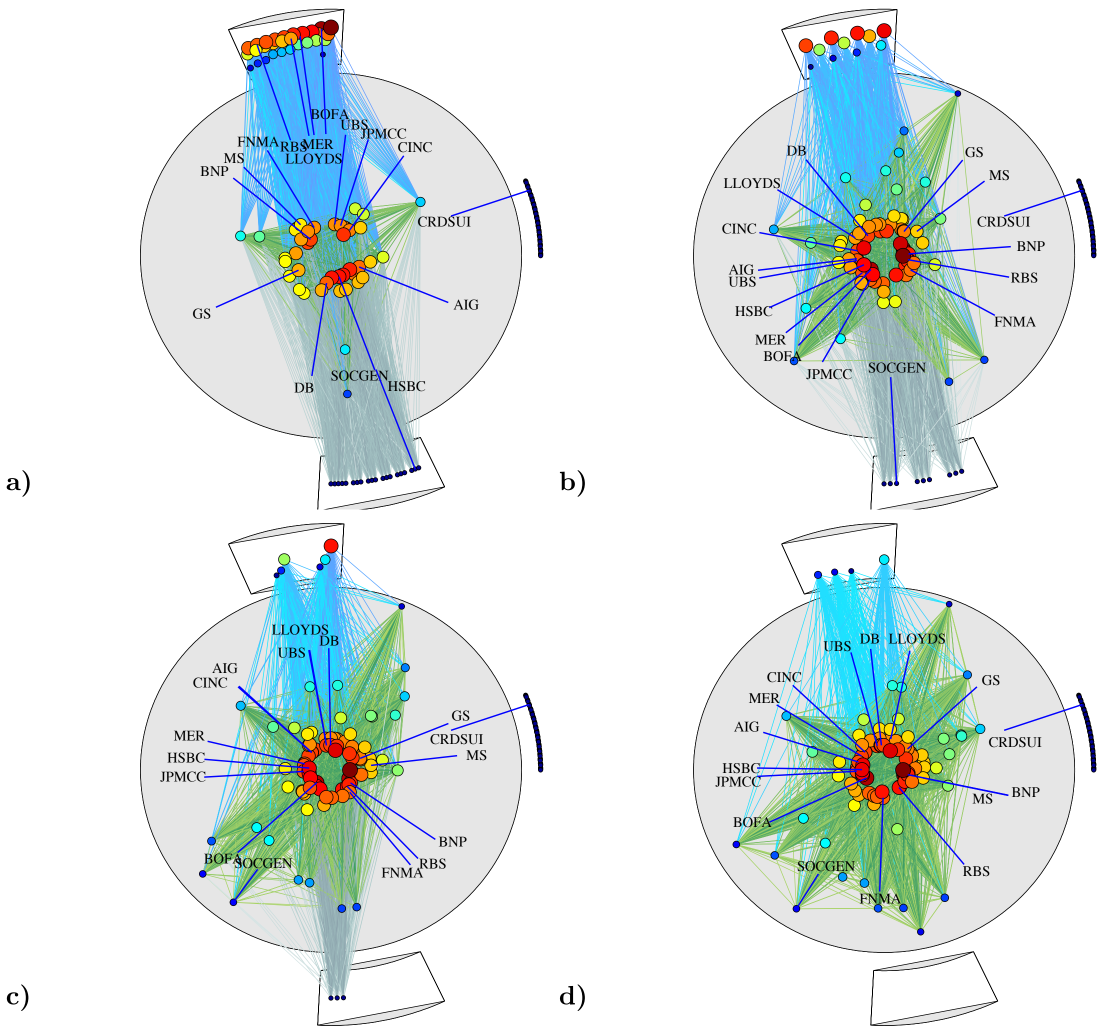

Figure S6. Bow-tie structure from period 1 for varying values of impacting-vulnerability centralities a) The bow-tie is constructed as in Fig. S1. Nodes with $0.9 < r_i < 1.1$ are in the SCC, nodes with $r_i > 1.1$ are in the IN, and the nodes with $r_i < 0.9$ are in the OUT **b)** The bow-tie is constructed as in Fig. S1. Nodes with $0.8 < r_i < 1.2$ are in the SCC, nodes with $r_i > 1.2$ are in the IN, and the nodes with $r_i < 0.8$ are in the OUT **c)** The bow-tie is constructed as in Fig. S1. Nodes with $0.7 < r_i < 1.3$ are in the SCC, nodes with $r_i > 1.3$ are in the IN, and the nodes with $r_i < 0.7$ are in the OUT **d)** The bow-tie is constructed as in Fig. S1. Nodes with $0.7 < r_i < 1.3$ are in the SCC, nodes with $r_i > 1.3$ are in the IN, and the nodes with $r_i < 0.7$ are in the OUT

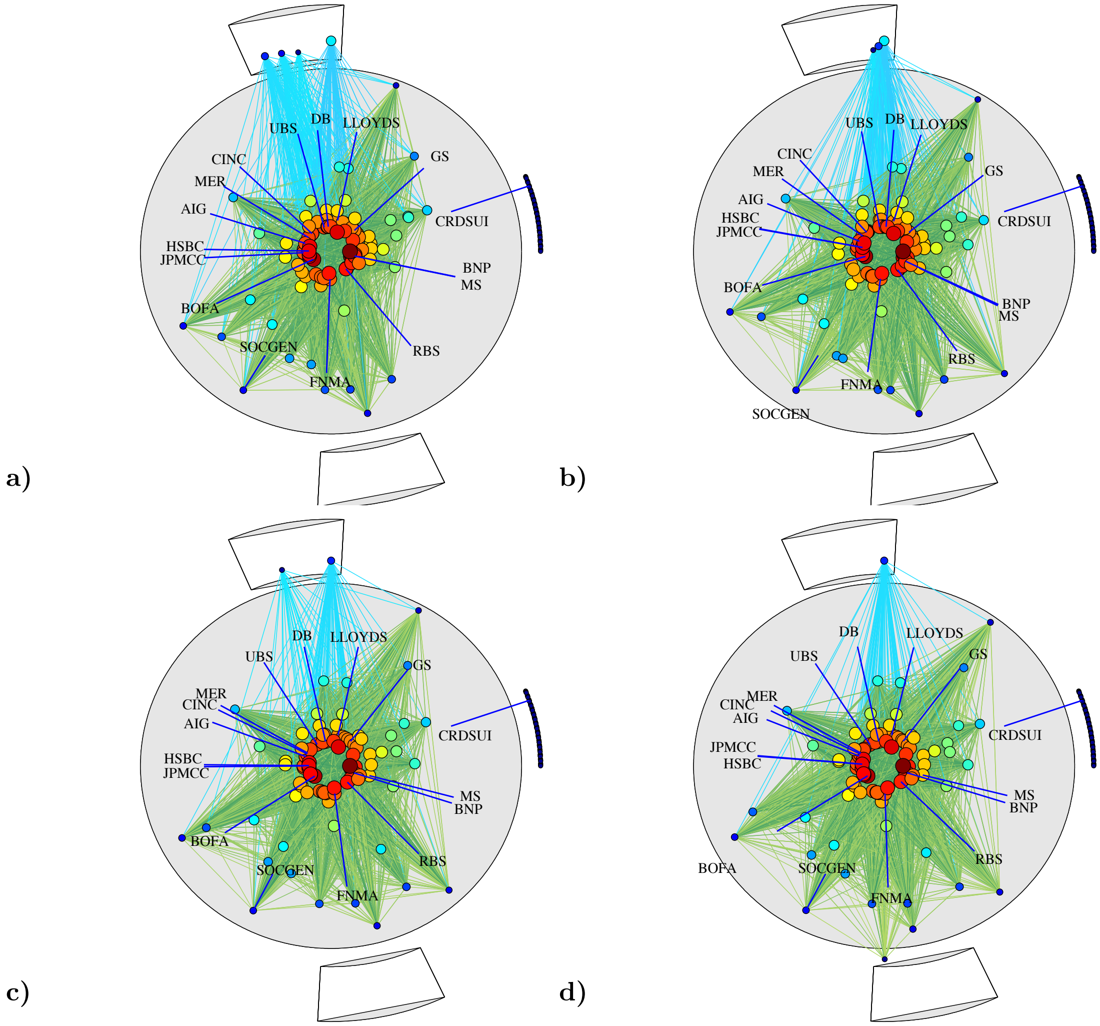

Figure S7. Bow-tie structure from period 1 for varying values of impacting-vulnerability centralities a) The bow-tie is constructed as in Fig. S1. Nodes with $0.5 < r_i < 1.5$ are in the SCC, nodes with $r_i > 1.5$ are in the IN, and the nodes with $r_i < 0.5$ are in the OUT **b)** The bow-tie is constructed as in Fig. S1. Nodes with $0.4 < r_i < 1.6$ are in the SCC, nodes with $r_i > 1.6$ are in the IN, and the nodes with $r_i < 0.4$ are in the OUT **c)** The bow-tie is constructed as in Fig. S1. Nodes with $0.3 < r_i < 1.7$ are in the SCC, nodes with $r_i > 1.7$ are in the IN, and the nodes with $r_i < 0.3$ are in the OUT **d)** The bow-tie is constructed as in Fig. S1. Nodes with $0.2 < r_i < 1.8$ are in the SCC, nodes with $r_i > 1.8$ are in the IN, and the nodes with $r_i < 0.2$ are in the OUT

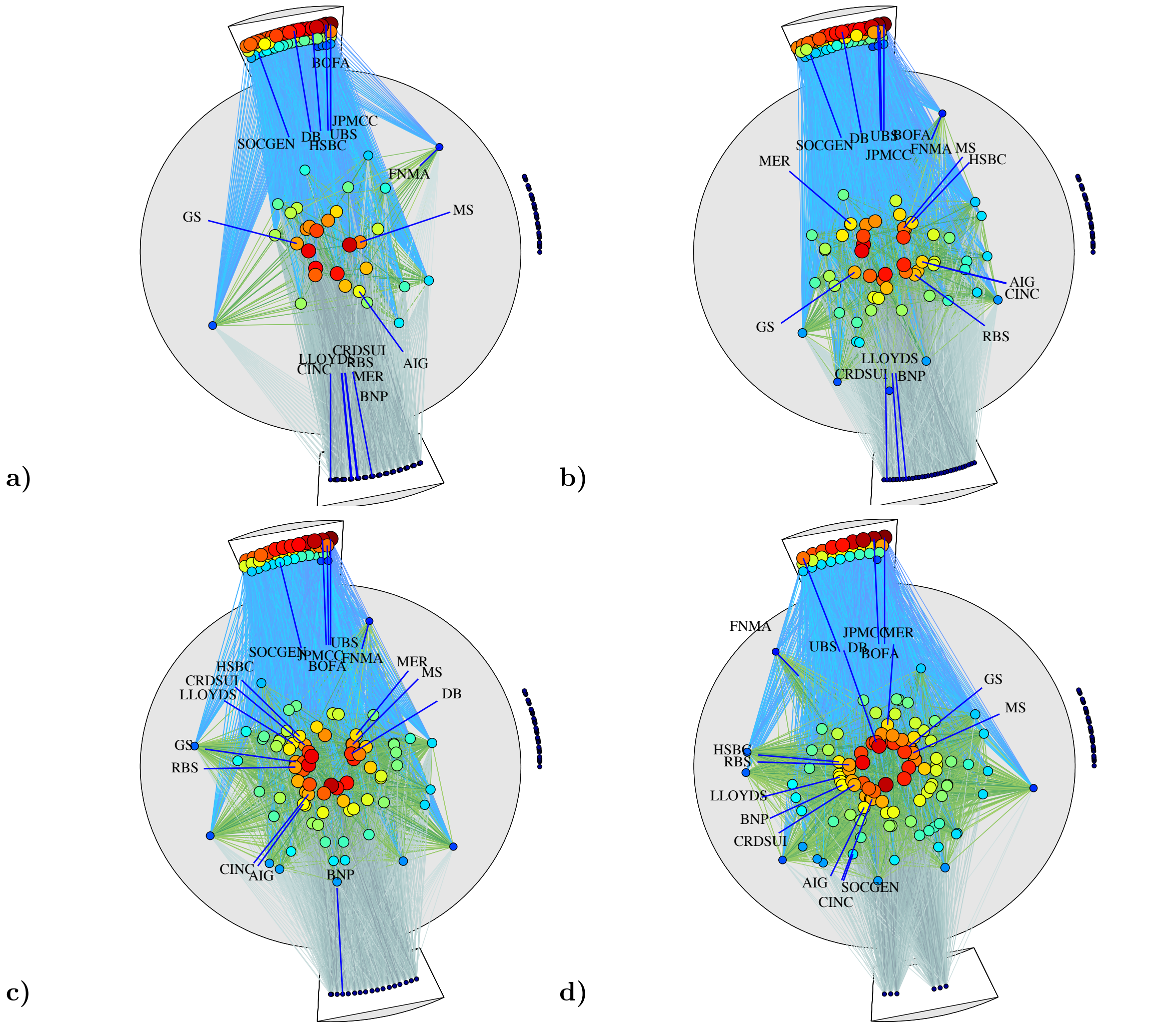

Figure S8. Bow-tie structure from period 2 for varying values of impacting-vulnerability centralities a) The bow-tie is constructed as in Fig. S1. Nodes with $0.9 < r_i < 1.1$ are in the SCC, nodes with $r_i > 1.1$ are in the IN, and the nodes with $r_i < 0.9$ are in the OUT **b)** The bow-tie is constructed as in Fig. S1. Nodes with $0.8 < r_i < 1.2$ are in the SCC, nodes with $r_i > 1.2$ are in the IN, and the nodes with $r_i < 0.8$ are in the OUT **c)** The bow-tie is constructed as in Fig. S1. Nodes with $0.7 < r_i < 1.3$ are in the SCC, nodes with $r_i > 1.3$ are in the IN, and the nodes with $r_i < 0.7$ are in the OUT **d)** The bow-tie is constructed as in Fig. S1. Nodes with $0.7 < r_i < 1.3$ are in the SCC, nodes with $r_i > 1.3$ are in the IN, and the nodes with $r_i < 0.7$ are in the OUT

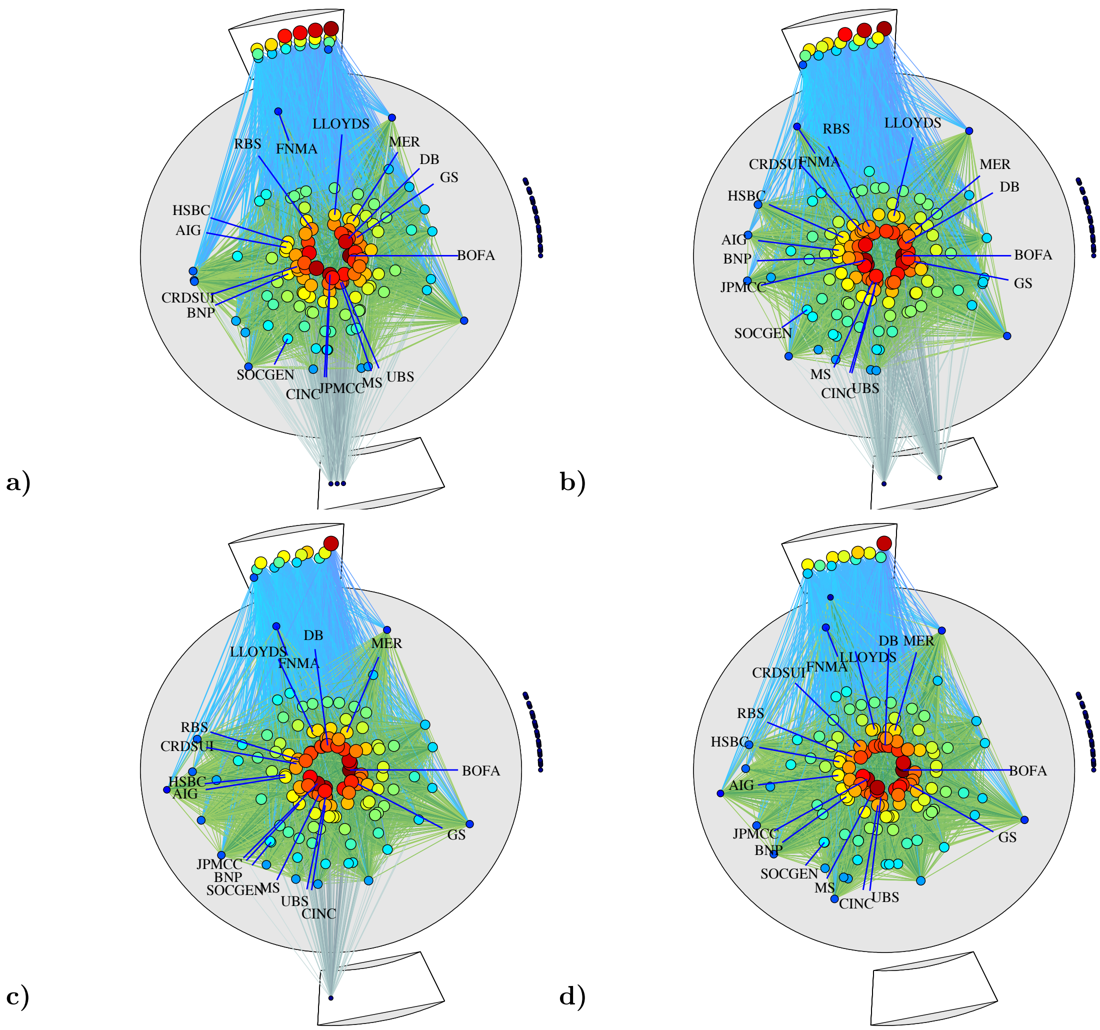

Figure S9. Bow-tie structure from period 2 for varying values of impacting-vulnerability centralities a) The bow-tie is constructed as in Fig. S1. Nodes with $0.5 < r_i < 1.5$ are in the SCC, nodes with $r_i > 1.5$ are in the IN, and the nodes with $r_i < 0.5$ are in the OUT **b)** The bow-tie is constructed as in Fig. S1. Nodes with $0.4 < r_i < 1.6$ are in the SCC, nodes with $r_i > 1.6$ are in the IN, and the nodes with $r_i < 0.4$ are in the OUT **c)** The bow-tie is constructed as in Fig. S1. Nodes with $0.3 < r_i < 1.7$ are in the SCC, nodes with $r_i > 1.7$ are in the IN, and the nodes with $r_i < 0.3$ are in the OUT **d)** The bow-tie is constructed as in Fig. S1. Nodes with $0.2 < r_i < 1.8$ are in the SCC, nodes with $r_i > 1.8$ are in the IN, and the nodes with $r_i < 0.2$ are in the OUT

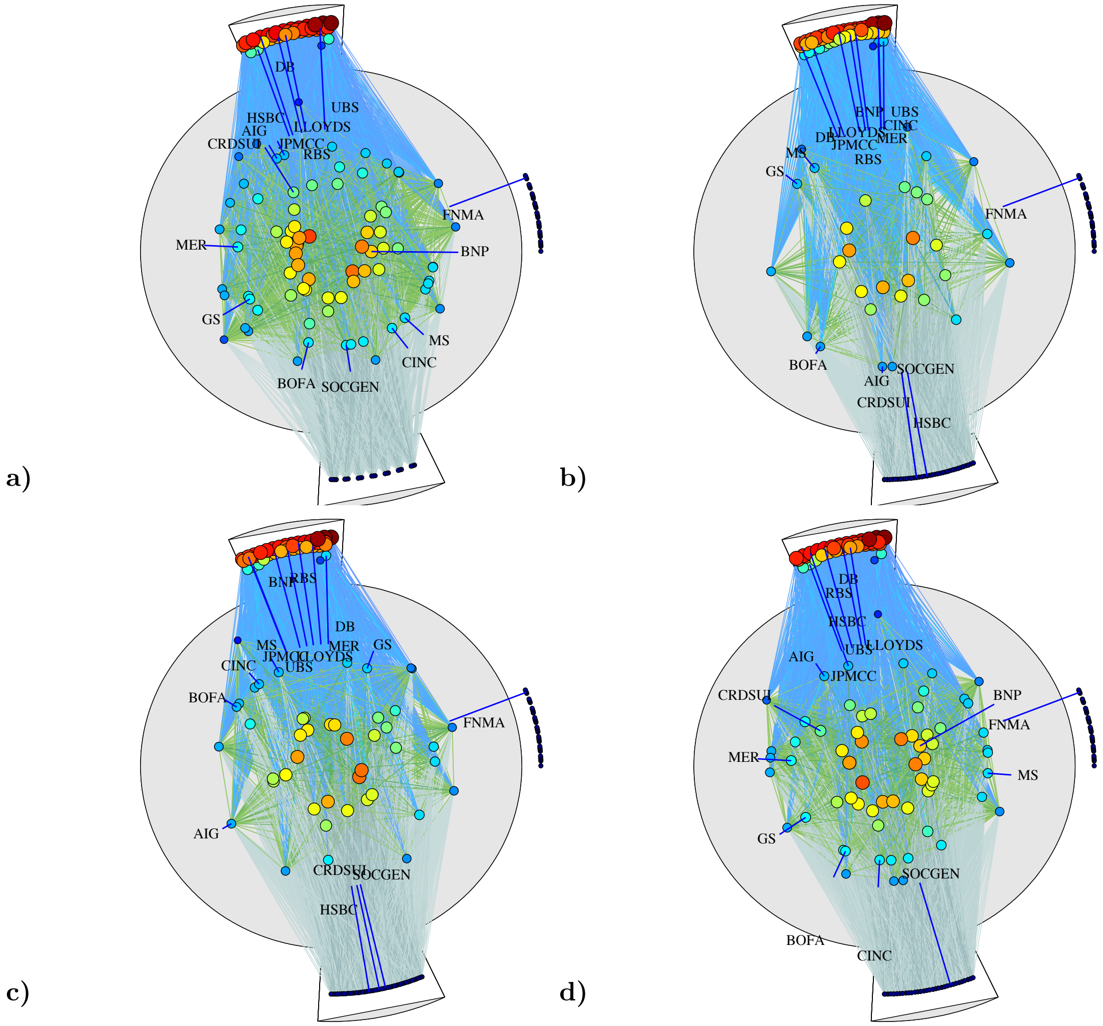

Figure S10. Bow-tie structure from period 3 for varying values of impacting-vulnerability centralities a) The bow-tie is constructed as in Fig. S1. Nodes with $0.5 < r_i < 1.5$ are in the SCC, nodes with $r_i > 1.5$ are in the IN, and the nodes with $r_i < 0.5$ are in the OUT **b)** The bow-tie is constructed as in Fig. S1. Nodes with $0.4 < r_i < 1.6$ are in the SCC, nodes with $r_i > 1.6$ are in the IN, and the nodes with $r_i < 0.4$ are in the OUT **c)** The bow-tie is constructed as in Fig. S1. Nodes with $0.3 < r_i < 1.7$ are in the SCC, nodes with $r_i > 1.7$ are in the IN, and the nodes with $r_i < 0.3$ are in the OUT **d)** The bow-tie is constructed as in Fig. S1. Nodes with $0.2 < r_i < 1.8$ are in the SCC, nodes with $r_i > 1.8$ are in the IN, and the nodes with $r_i < 0.2$ are in the OUT

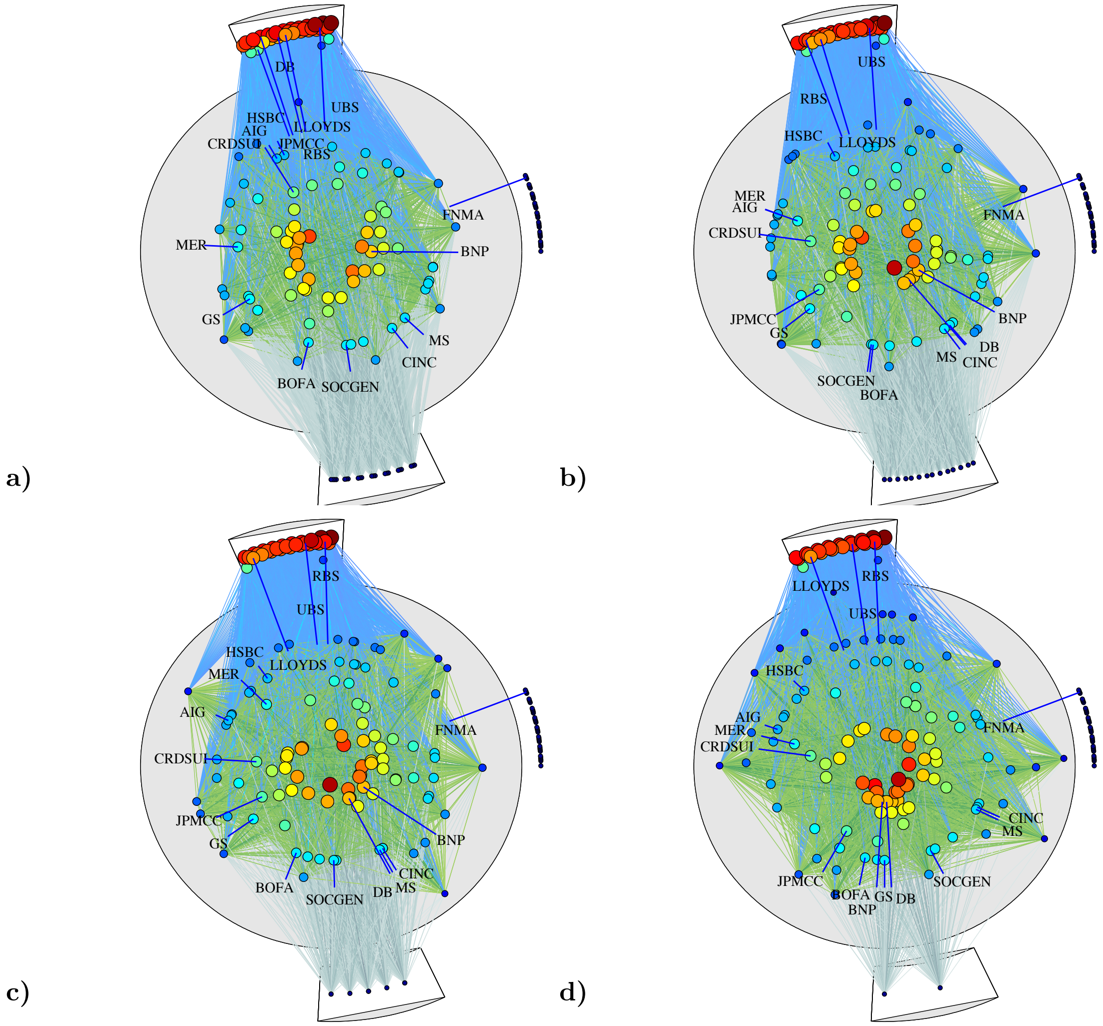

Figure S11. Bow-tie structure from period 3 for varying values of impacting-vulnerability centralities a) The bow-tie is constructed as in Fig. S1. Nodes with $0.5 < r_i < 1.5$ are in the SCC, nodes with $r_i > 1.5$ are in the IN, and the nodes with $r_i < 0.5$ are in the OUT **b)** The bow-tie is constructed as in Fig. S1. Nodes with $0.4 < r_i < 1.6$ are in the SCC, nodes with $r_i > 1.6$ are in the IN, and the nodes with $r_i < 0.4$ are in the OUT **c)** The bow-tie is constructed as in Fig. S1. Nodes with $0.3 < r_i < 1.7$ are in the SCC, nodes with $r_i > 1.7$ are in the IN, and the nodes with $r_i < 0.3$ are in the OUT **d)** The bow-tie is constructed as in Fig. S1. Nodes with $0.2 < r_i < 1.8$ are in the SCC, nodes with $r_i > 1.8$ are in the IN, and the nodes with $r_i < 0.2$ are in the OUT

Materials and Methods

Data

Credit Default Swaps (CDS's) are financial derivatives instruments in which the seller provides the buyer protection against a credit event of a reference entity Our aim is to analyse the time series data of CDS

prices, or spreads, of top US and European financial institutions in the last years. The data, acquired via a subscription to Bloomberg, consists of CDS spreads of single name entities denominated in US dollars and in the Euro, encompassing a total of 176 top firms in the financial sector, in the period from 2nd January 2002 until 1st December 2011. As shown in Fig. S12, the time series display three distinct phases. Accordingly, we divide the data into three parts: (1) January 2002 - May 2006 (representative of a normal phase); (2) May 2006 - March 2009 (volatile with an upwards trend); (3) March 2009 - December 2011 (volatile with a downwards trend market scenario). The motivation to do a period-wise analysis is to extract the network structure before, during and after the crisis of 2008. This data window covers a 2560 weekdays.

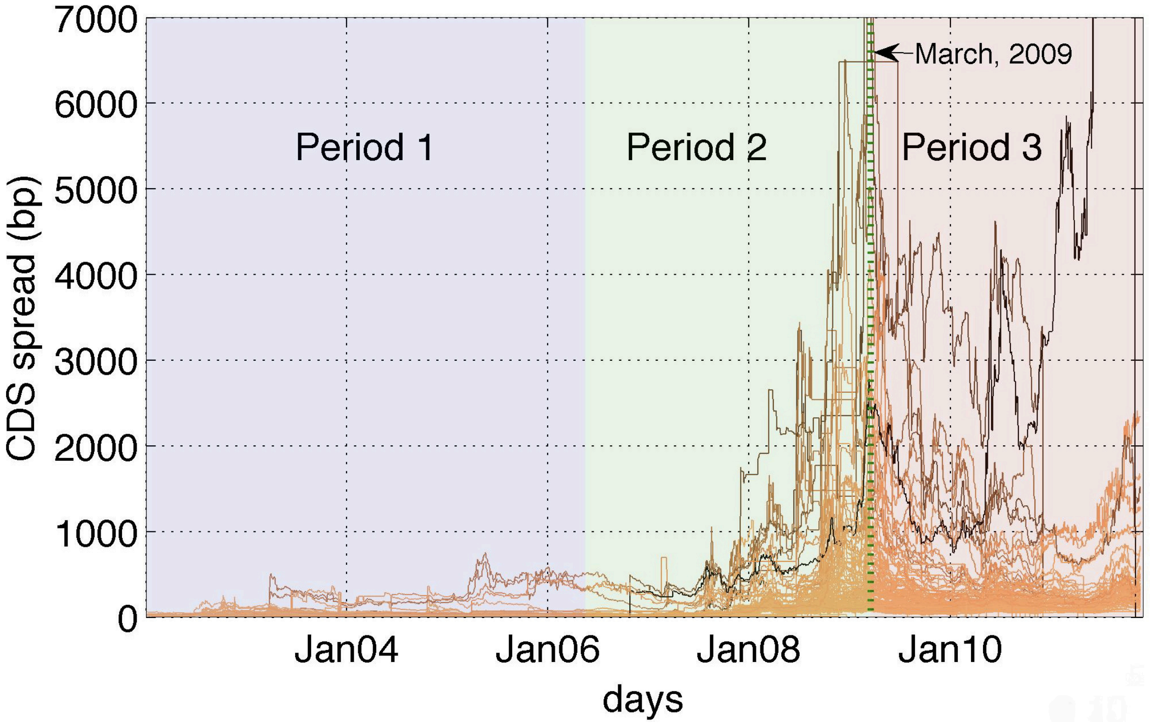

Figure S12. Time series of credit default swaps throughout the credit crisis. A plot of the CDS spread time series covering the financial crisis of 2008. The data ranges from January 2002 to December 2011. We can observe three market phases. Most CDS spreads peak around March 2009. The CDS prices are quoted in basis points (bp). The purpose of this plot is to highlight the market regimes, rather than the individual CDS spread evolution. Accordingly, the CDS spreads of all the financial entities are plotted here.

References

References

1. Battiston S, Glattfelder JB, Garlaschelli D, Lillo F, Caldarelli G (2010) The Structure of Financial Networks. Network Science : 131–163.

2. Page L, Brin S, Motwani R, Winograd T (1998) The PageRank citation ranking: Bringing order to the web. Technical report, Stanford Digital Library Technologies Project.

3. FCIC FCIC (2011) The financial crisis inquiry report: final report of the National Commission on the Causes of the Financial and Economic Crisis in the United States. Public Affairs.
